# Diagnostic Challenges in *ABCA4*-Associated Retinal Degeneration: One Gene, Many Phenotypes

**DOI:** 10.3390/diagnostics13233530

**Published:** 2023-11-25

**Authors:** Tien-En Tan, Rachael Wei Chao Tang, Choi Mun Chan, Ranjana S. Mathur, Beau J. Fenner

**Affiliations:** 1 Singapore National Eye Centre, Singapore Eye Research Institute, Singapore 168751, Singapore; tantienen@gmail.com (T.-E.T.);; 2 Ophthalmology & Visual Sciences Academic Clinical Programme (EYE ACP), Duke-NUS Medical School, Singapore 169857, Singapore

**Keywords:** *ABCA4*, Stargardt disease, inherited retinal disease, retinal dystrophy, phenotypic variation, genetic diagnosis

## Abstract

(1) Purpose: *ABCA4*-associated retinal degeneration (*ABCA4*-RD) is a phenotypically diverse disease that often evades diagnosis, even by experienced retinal specialists. This may lead to inappropriate management, delayed genetic testing, or inaccurate interpretation of genetic testing results. Here, we illustrate the phenotypic diversity of *ABCA4*-RD using a series of representative cases and compare these to other conditions that closely mimic *ABCA4*-RD. (2) Methods: Genetically confirmed *ABCA4*-RD cases with representative phenotypes were selected from an inherited retinal disease cohort in Singapore and compared to phenocopies involving other retinal diseases. (3) Results: *ABCA4*-RD phenotypes in this series included typical adolescent-onset Stargardt disease with flecks, bull’s eye maculopathy without flecks, fundus flavimaculatus, late-onset Stargardt disease, and severe early-onset Stargardt disease. Phenocopies of *ABCA4*-RD in this series included macular dystrophy, pattern dystrophy, cone dystrophy, advanced retinitis pigmentosa, Leber congenital amaurosis, drug toxicity, and age-related macular degeneration. Key distinguishing features that often suggested a diagnosis of *ABCA4*-RD were the presence of peripapillary sparing, macular involvement and centrifugal distribution, and a recessive pedigree. (4) Conclusions: *ABCA4*-RD demonstrates a remarkable phenotypic spectrum that makes diagnosis challenging. Awareness of the clinical spectrum of disease can facilitate prompt recognition and accurate diagnostic testing.

## 1. Introduction

Inherited retinal diseases (IRDs) are a heterogenous group of monogenic conditions that may affect only the retina or also involve other body systems as part of a syndrome, with an estimated overall prevalence of about 1 in 3000 worldwide [1,2,3]. Although still considered orphan diseases, IRDs as a group have become the leading cause of legal blindness among working age adults in developed nations [4,5]. IRDs present a particular diagnostic challenge because of their broad genetic and phenotypic diversity. More than 250 different genetic loci have been implicated in monogenic IRD, which cause a wide range of possible phenotypes, such as retinitis pigmentosa (RP), cone or cone–rod dystrophy (CORD), various macular dystrophies, and choroideremia, to name but a few [6,7,8]. A universal classification system of IRDs has proved elusive, but some proposed phenotypic classification systems have up to 62 diagnostic categories [7]. Compounding the diagnostic problem is that variants in a single gene can cause multiple phenotypes, and conversely, a single phenotype can also be caused by variants in many different genes. Adding to the complexity of the problem is the fact that the phenotypic appearance of IRDs can also overlap significantly with retinal disease from non-monogenic or acquired etiologies, such as inflammatory, infective, toxic, or age-related retinal degenerations [9,10,11,12].

Given the diagnostic complexity of IRDs, and the scarcity of dedicated sub-specialty IRD expertise in many regions around the world, many IRD patients experience long diagnostic odysseys, and are frequently given multiple different diagnoses or are misdiagnosed. Erroneous diagnoses such as age-related macular degeneration may lead to patients receiving unnecessary or even potentially harmful treatments. More importantly, accurate diagnosis of the underlying genetic cause of an IRD allows for proper genetic counselling, and allows patients appropriate access to therapeutic trials. The first gene therapy for an IRD received regulatory approval in 2017, and is likely to be followed in the coming years by other gene-based therapies, that are likely to be genotype-specific [6,13,14]. The anticipated availability of therapeutic options for IRDs that are contingent on the specific underlying genetic cause makes accurate genetic diagnosis more crucial than ever for the millions of individuals living with IRDs worldwide [15].

Pathogenic mutations in *ABCA4* are the commonest cause of IRD worldwide, with the estimated population carrier frequency of certain mild disease-causing *ABCA4* variants being as high as 1 in 20 [7,8,16,17]. *ABCA4*-associated retinal degeneration (*ABCA4*-RD) exhibits significant phenotypic variability. Mutations in *ABCA4* can cause a wide spectrum of IRD phenotypes, including Stargardt disease, fundus flavimaculatus, bull’s eye maculopathy, CORD, and an RP-like phenotype, among others [16]. The high prevalence of *ABCA4*-RD combined with its protean phenotypic manifestations that mimic other IRDs and non-monogenic retinal diseases make it a frequently misdiagnosed condition in clinical practice [18]. In this study, we present a series of representative *ABCA4*-RD cases from a prospectively enrolled IRD cohort, and compare them with a series of phenocopies that often mimic *ABCA4*-RD, to highlight key distinguishing features that can aid in accurate diagnosis.

## 2. Materials and Methods

This was a case series selected from a cohort of IRD patients from the Singapore National Eye Centre (SNEC), Singapore. Genetically confirmed *ABCA4*-RD cases with representative phenotypes were selected, as well as associated retinal conditions that were phenocopies mimicking *ABCA4*-RD. *ABCA4*-RD cases and phenocopies were compared, and distinguishing features to aid in accurate diagnosis were identified.

Clinical diagnoses were made by fellowship-trained retinal specialists based on a combination of clinical history and examination, multimodal imaging, as well as psychophysical and electrophysiologic testing where required. Genetic diagnoses were made through multi-disciplinary team (MDT) meetings involving fellowship-trained retinal specialists, geneticists, bioinformaticians and genetic counsellors. This study was approved by the SingHealth Institutional Review Board (IRB) and was carried out in accordance with the tenets of the Declaration of Helsinki. Informed consent or a waiver of consent from the IRB (for use of anonymized data) was obtained from all participants.

Clinical phenotyping was performed in dedicated IRD clinics, which included clinical history taking, visual acuity (VA) assessment with logMAR testing with pinhole or manifest refraction, color vision assessment with Ishihara pseudoisochromatic plates (where indicated), intraocular pressure measurement by tonometry, and clinical examination with slit-lamp biomicroscopy and indirect ophthalmoscopy. Imaging investigations included macula-centered 45° to 50° color fundus photography, ultrawidefield (UWF) pseudocolor and pseudocolor photography (Optos California, Optos, Dunfermline, UK; Clarus 500, Carl Zeiss Meditec AG, Jena, Germany), 30° to 55° and UWF autofluorescence (AF) imaging (Spectralis, Heidelberg Engineering, Heidelberg, Germany; Optos California, Optos) and macula-centered optical coherence tomography (OCT; Spectralis, Heidelberg). Pscyhophysical testing was performed by Goldmann kinetic perimetry (Haag-Streit, Koeniz, Switzerland), and electroretinography (ERG) was performed where required according to ISCEV Standard methodology [19]. Non-IRD phenocopy cases underwent similar clinical evaluations and multimodal imaging as indicated.

Genetic testing was performed on DNA obtained from peripheral blood leukocytes as per standard procedures. Whole exome sequencing (WES) was performed by Macrogen (Geumcheon-gu, Seoul, Republic of Korea). Where required, direct sequencing was performed using the BigDye Terminator Cycle Sequencing kit (Applied Biosystems, Foster City, CA, USA) with 10 ng of template DNA in each reaction and a PCR program of 25 cycles of denaturation at 97 °C for 30 s, annealing at 50 °C for 15 s, and extension at 60 °C for 4 min. Samples were analyzed in a 3130 Genetic Analyzer (Applied Biosystems). Bioinformatics analysis of the raw sequence data was performed at the SingHealth Duke-NUS Institute of Precision Medicine (PRISM), Singapore. Genetic variant calls were made by comparison against SG10K, a Singapore population-based genome reference database [20]. Determination of variant pathogenicity was based on established American College of Medical Genetics and Genomics (ACMG) criteria [21]. The gnomAD database was queried to identify *ABCA4* and other variants classified as pathogenic or likely pathogenic, according to annotations from the ClinVar database [22,23]. In cases where two or more causative variants of *ABCA4* or other recessive genes were detected, segregation analysis was performed where feasible to establish phase.

## 3. Results

Six representative *ABCA4*-RD cases were selected (Cases 1a to 6a), along with seven associated phenocopy cases (Cases 1b to 6b, and 2c). The phenotypic characteristics and information on genetic variants for the 6 *ABCA4*-RD cases are summarized in Table 1. All *ABCA4*-RD cases in this series had at least two causative *ABCA4* variants identified. We highlight each representative *ABCA4*-RD case alongside its phenocopies, to highlight salient distinguishing features for accurate diagnosis (Table 2).

### 3.1. Cases 1a versus 1b: Typical Adolescent-Onset Stargardt Disease with Macular Atrophy, Flecks, and Peripapillary Sparing Versus PRPH2-Associated Macular Dystrophy

**Figure 1:**
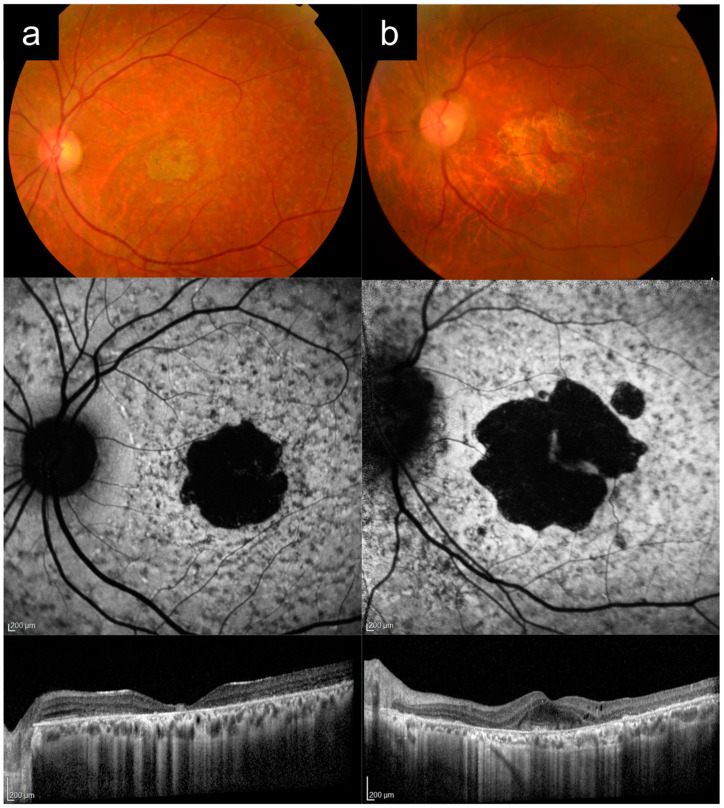
Cases 1a versus 1b: Typical adolescent-onset Stargardt disease with macular atrophy, flecks and peripapillary sparing (**a**) versus *PRPH2*-associated macular dystrophy (**b**). Note the similar appearance on autofluorescence imaging, except for the peripapillary sparing, which is present in Case 1a, but absent in Case 1b. These cases can also be distinguished clinically based on age of onset and inheritance patterns.

Case 1a presented with bilateral blurred central vision at 15 years of age, which progressively worsened over time. There were no other affected family members, consistent with autosomal recessive (AR) inheritance. Figure 1a shows multimodal imaging from when the patient was in his 30s. VA in the left eye at that time was 6/45. Fundus examination and CFP imaging showed an area of foveal retinal pigment epithelium (RPE) atrophy with a granular appearance and metallic sheen, surrounded by yellow pisciform flecks distributed throughout the posterior pole and out to the mid-peripheral retina. AF imaging showed a central geographic patch of hypo-AF surrounded by flecks with mixed hyper- and hypo-AF signal. Note the typical sparing of the peripapillary retina, which is more obvious on AF imaging, but also visible on CFP, albeit more subtle. OCT imaging showed outer retinal atrophy and ellipsoid zone (EZ) loss, with patchy choroidal hypertransmission, again with notable peripapillary sparing. Genetic testing revealed two likely pathogenic *ABCA4* variants in this individual (Table 1).

Case 1b presented with complaints of persistent blurred vision and mild metamorphopsia in the left eye after uneventful cataract surgery in his late 60s. Best-corrected VA (BCVA) by manifest refraction was 6/7.5. Figure 1b shows imaging from this time, after his cataract surgery. CFP imaging showed a broad annulus of perifoveal RPE atrophy, with sparing of a small foveal island (explaining the good vision). In the rest of the macula, there were areas of subtle RPE atrophic change, but no frank flecks or yellow subretinal deposits. AF imaging, however, revealed much more apparent fleck-like lesions, although there was no peripapillary sparing. OCT imaging showed perifoveal RPE and outer retinal atrophy, with relative sparing of a small subfoveal region. Prior to genetic testing results, this patient had been diagnosed by multiple specialists as having “late onset Stargardt disease”. Genetic testing subsequently identified the *PRPH2* c.533A>G p.Q178R variant. Pedigree by history revealed that the subject’s mother was also likely affected, which was consistent with an autosomal-dominant (AD) pattern of inheritance.

Case 1a represents a typical phenotype of *ABCA4*-RD, presenting with adolescent-onset “classical” Stargardt disease with macular atrophy, flecks, and peripapillary sparing [16,24,25]. Case 1b is a *PRPH2*-associated macular dystrophy mimicking Stargardt disease. Key distinguishing features between these two cases are the age of onset, peripapillary sparing, and the inheritance pattern from the pedigree (Table 2). The younger age of onset in adolescence, presence of clear peripapillary sparing, and the AR inheritance pattern all favor a diagnosis of *ABCA4*-RD versus *PRPH2*-RD. Note that the yellow deposits or flecks and AF appearance can look very similar in both diseases, as in this case. In addition, the small area of central foveal sparing in Case 1b, though absent in this Case 1a, can also occur in *ABCA4*-RD, as in Case 4a later.

### 3.2. Cases 2a versus 2b versus 2c: Stargardt Disease without Flecks Versus GUCY2D-Associated Cone Dystrophy versus Hydroxychloroquine Toxicity

**Figure 2:**
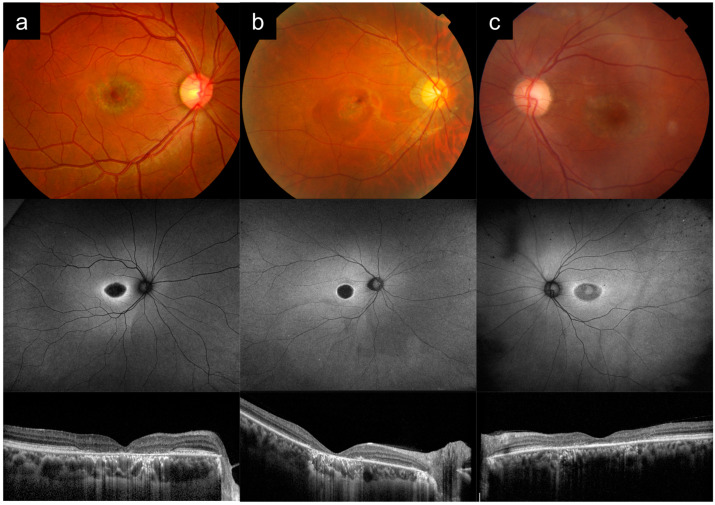
Cases 2a versus 2b versus 2c: Stargardt disease without flecks (**a**) versus *GUCY2D*-associated cone dystrophy (**b**) versus hydroxychloroquine toxicity (**c**). Note the similar appearance between Cases 2a and Case 2b, particularly on autofluorescence and optical coherence tomography imaging. The slight metallic sheen within the area of atrophy on the color photograph in Case 2a points towards *ABCA4*-associated Stargardt disease. These cases can also be distinguished based on clinical symptoms, electroretinography, and inheritance patterns. Case 2c shows that systemic drug toxicity can present with similar imaging features that mimic inherited retinal disease.

Case 2a presented with difficulty reading at 20 years old. There were no other affected family members, which was consistent with AR inheritance. Figure 2a shows multimodal imaging from the right eye in his 20s. VA in the right eye varied from 6/12 to 6/15 at the time. CFP imaging demonstrates a central area of RPE atrophy at the fovea, with a slight metallic sheen, but without flecks. AF imaging shows a central oval hypo-AF region corresponding to the foveal atrophy, surrounded by a hyper-AF ring, and similarly confirms the absence of flecks. OCT imaging shows a central area of RPE and outer retinal atrophy. ERG showed mainly macular dysfunction, with mild/borderline reduction in full-field cone responses and normal rod function. Genetic testing revealed an *ABCA4* genotype (Table 1).

Case 2b presented with poor vision noted in school around 10 years old, with progressive hemeralopia and glare. BCVA in his 20s was 6/60 and 6/45 in the right and left eyes, respectively. Pedigree analysis revealed 6 other affected family members in two generations, with male-to-male transmission, indicating AD inheritance. Figure 2b shows imaging from his 20s. CFP imaging shows a central area of RPE atrophy. AF imaging shows a central round hypo-AF region corresponding to the area of atrophy surrounded by a brighter hyper-AF ring. OCT imaging shows a central area of RPE and outer retinal atrophy. ERG showed significant reduction in full-field cone responses and macular function, with normal/borderline rod responses. Genetic testing identified the pathogenic *GUCY2D* c.2512c>T p.R838C allele.

Case 2c presented with blurred vision at 62 years old. She had no family history of ocular disease, but she a history of rheumatoid arthritis, being treated with hydroxychloroquine for more than 10 years. She was diagnosed with hydroxychloroquine toxicity and the medication was stopped. Figure 2c shows images from the left eye when she was 72 years old. VA in that eye was 6/15 at the time. CFP imaging shows an area of foveal RPE atrophy in a bull’s eye pattern. AF imaging shows a bull’s eye pattern with central hyper-AF, surrounded by a zone of hypo-AF, and an outside hyper-AF ring. OCT imaging shows perifoveal outer retinal atrophy, with some relative sparing of the subfoveal region.

Case 2a demonstrates *ABCA4*-RD with a phenotype of Stargardt disease without flecks, or bull’s eye maculopathy, which can be easily mistaken for cone dystrophy, such as the phenotype of Case 2b, which was a *GUCY2D*-associated AD cone dystrophy. Note that the appearance on imaging for both cases are very similar, especially on AF and OCT. Key distinguishing features between Cases 2a and 2b are the clinical symptoms, appearance of the atrophy, ERG findings, and inheritance patterns (Table 2). Prominent symptoms of glare and hemeralopia, significantly diminished full-field cone responses, and the AD inheritance pattern in Case 2b point towards AD cone dystrophy, rather than *ABCA4*-associated Stargardt disease. Case 2c shows that systemic drug toxicity is another important cause of bull’s eye maculopathy and can present with similar imaging features that mimic IRD. This serves as an important reminder that drug toxicity must be excluded in all such cases.

### 3.3. Cases 3a versus 3b: Fundus Flavimaculatus versus Autosomal-Dominant Pattern Dystrophy

**Figure 3:**
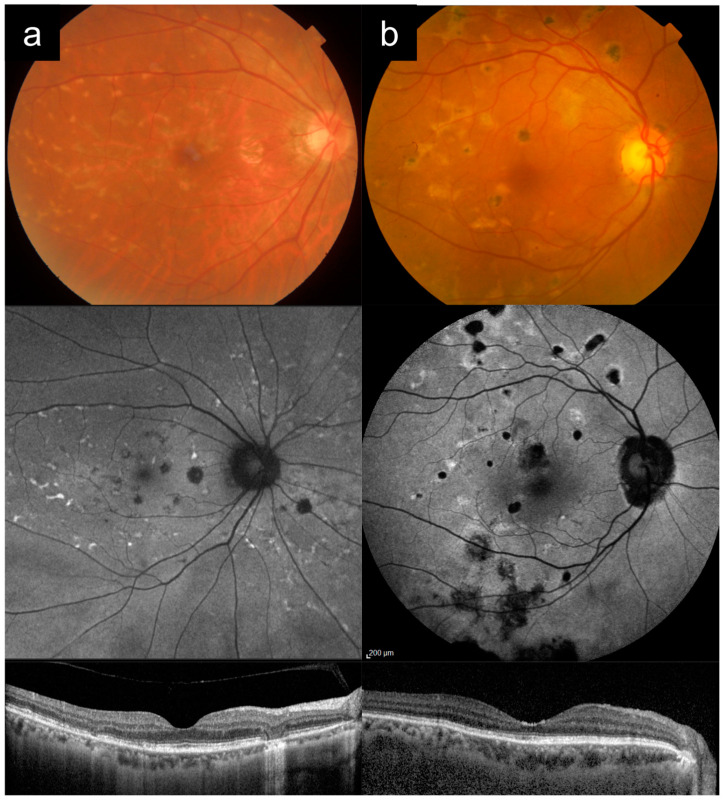
Cases 3a versus 3a: Fundus flavimaculatus (**a**) versus autosomal-dominant pattern dystrophy (**b**). These cases both presented with late onset, yellow flecks, good vision, and minimal macular atrophy. Note the peripapillary sparing on autofluorescence imaging in Case 3a that points towards *ABCA4*-associated retinal degeneration. Also note the prominent pigment clumps within the yellow flecks in Case 3b, which are more characteristic of *PRPH2*-associated pattern dystrophy, although genetic testing results were not available for this case. These cases can also be distinguished based on the inheritance patterns.

Case 3a first presented at 57 years old, asymptomatic, with an incidental finding of yellow flecks in the posterior pole bilaterally. VA was 6/6 in both eyes at the time. She had no other affected members of the family. Figure 3a shows imaging from the right eye in her early 60s. VA was 6/9.5 in both eyes at the time. CFP imaging shows yellow pisciform flecks in the posterior pole, sparing the fovea and with a reticular pattern temporally. There is a round patch of RPE atrophy along the papillomacular bundle. AF imaging shows that the flecks are mixed hypo- and hyper-AF and demonstrates peripapillary sparing. OCT imaging shows patchy outer retinal atrophy, with foveal preservation. Genetic testing demonstrated two causative *ABCA4* variants in this patient (Table 1).

Case 3b presented with progressive blurring of vision from 46 years of age. She was found to have yellow flecks with hyperpigmentation in both eyes. Her father and grandfather were similarly affected, consistent with an AD inheritance pattern. Figure 3b shows imaging of the right eye taken in her 60s and 70s. VA in this eye was 6/7.5. CFP imaging showed broad yellow flecks throughout the posterior pole in a reticular pattern, with clumps of pigment within. AF imaging demonstrates that the flecks are mostly hyper-AF, with hypo-AF signal from the pigment clumps. OCT shows mild subfoveal EZ disruption, but otherwise relatively well-preserved outer retina in this slice. Genetic testing results were not available for this patient.

This pair of cases highlights a mild, late-onset phenotype of *ABCA4*-RD, with predominantly flecks and no foveal atrophy, which is frequently described as fundus flavimaculatus, and which closely mimics pattern dystrophy. Peripapillary sparing is well demonstrated on AF imaging in this case, which points towards *ABCA4*-RD, but this can also sometimes be seen in pattern dystrophy. Case 3b is a case of AD pattern dystrophy, and although no genetic testing results were available for this patient, a large proportion of such cases are related to *PRPH2* mutations. A key distinguishing feature in this case would be the presence of prominent pigment clumps within large yellow flecks, which is more characteristic of *PRPH2*-related disease. The AR pedigree in Case 3a and the AD pedigree in Case 3b also help to distinguish the two different causes in this pair, but this is not always reliable. *PRPH2*-associated pattern dystrophy can show variable penetrance with mild or late-onset disease that may obscure a dominant inheritance pattern, and *ABCA4*-RD can exhibit pseudodominant inheritance too. In some cases, genetic testing is the only reliable way of differentiating *ABCA4*-RD from *PRPH2*-associated disease.

### 3.4. Cases 4a versus 4b: Late-Onset Stargardt Disease versus Age-Related Macular Degeneration (AMD) with Geographic Atrophy

**Figure 4:**
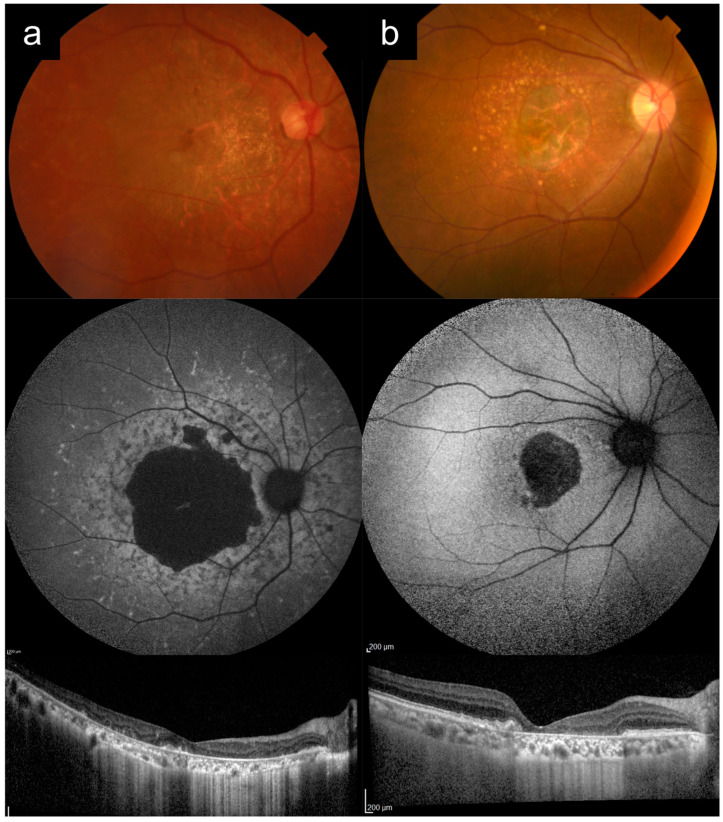
Cases 4a versus 4b: Late-onset Stargardt disease (**a**) versus dry age-related macular degeneration (AMD) with geographic atrophy (**b**). In Case 4a, the yellow flecks are more elongated and pisciform, and many more flecks are apparent on autofluorescence imaging where they are both hyper- and hypo-autofluorescent. There is also peripapillary sparing, with the nasal edge of the atrophy concave towards the disc as a result. In Case 4b, the yellow drusen are rounder, and do not have a significant autofluorescence signal. The nasal edge of the atrophy is convex towards the disc. There is a shallow irregular pigment epithelial detachment temporal to the atrophy on optical coherence tomography that is associated with AMD.

Case 4a presented at 51 years old, for abnormalities detected on her diabetic retinopathy screening photograph. VA was 6/6 in the right eye, and 6/7.5 in the left eye at the time. On questioning, she had symptoms of blurred central vision in both eyes from 49 years of age, and no known family history of ocular disease or IRD. Figure 4a shows imaging of her right eye in her 60 s. She had a large geographic area of RPE atrophy with a small subfoveal island of sparing visible on CFP and AF imaging. On CFP imaging, some yellow flecks are visible in the temporal macula. Hypo-AF and hyper-AF flecks were much more apparent on AF imaging, with peripapillary sparing. OCT showed extensive RPE and outer retinal atrophy in the macula, with a small central island of relative sparing. Genetic testing revealed two pathogenic *ABCA4* missense variants (Table 1).

Case 4b presented at 69 years old with bilateral blurred vision. Figure 4b shows imaging from her right eye taken at 77 years old. VA was 6/60 in the right eye. The CFP image shows a large area of geographic atrophy involving the foveal center, surrounded by small- to medium-hard drusen. AF imaging shows hypo-AF corresponding to the area of geographic atrophy, surrounded by a band-like pattern of hyper-AF in the junctional zone, and some patchy hyper-AF signal further nasally, not sparing the peripapillary area. OCT showed a confluent area of complete RPE and outer retinal atrophy, and a temporal shallow irregular PED.

This pair of cases helps to highlight the differences between late-onset Stargardt disease (Case 4a) and age-related macular degeneration (AMD) with geographic atrophy (Case 4b). This is not an uncommon diagnostic dilemma in patients presenting in their 50s or 60s with bilateral, symmetrical patches of RPE atrophy, and some yellow deposits. Pedigree analysis may not be as helpful in these older age groups, or when dealing with late-onset disease, and an absence of affected family members is also not uncommon in AR inheritance. Some of the key distinguishing features are as follows: (1) the yellow flecks in *ABCA4*-RD are typically more elongated than round, and often meet at obtuse angles, creating pisciform shapes or a more extensive branching or reticular pattern, whereas the yellow drusen in AMD are typically more round (as in Case 4b); (2) AF imaging can be very useful, especially if the flecks are subtle or already atrophic, and can often reveal many more flecks than are apparent on clinical examination or CFP, and provide better appreciation of their pattern (as in Case 4a); (3) flecks in *ABCA4*-RD are usually hyper-AF (early on) or hypo-AF (later on), whereas drusen do not typically show up with frank AF abnormality; (4) peripapillary sparing, if present, points towards *ABCA4*-RD. Because of peripapillary sparing, the nasal edge of the RPE atrophy in Case 4a is concave towards the disc, while the nasal edge of the RPE atrophy in Case 4b is convex towards the disc; (5) the presence of a small island of central foveal sparing surrounded by an annular area of atrophy (as in Case 4a) is more suggestive of *ABCA4*-RD, although this can occasionally be seen in extrafoveal geographic atrophy from AMD; (6) the presence of a shallow irregular PED adjacent to the area of atrophy in Case 4b is associated with AMD rather than *ABCA4*-RD.

### 3.5. Cases 5a versus 5b: Severe, Early-Onset Stargardt Disease versus Late-Stage Retinitis Pigmentosa

**Figure 5:**
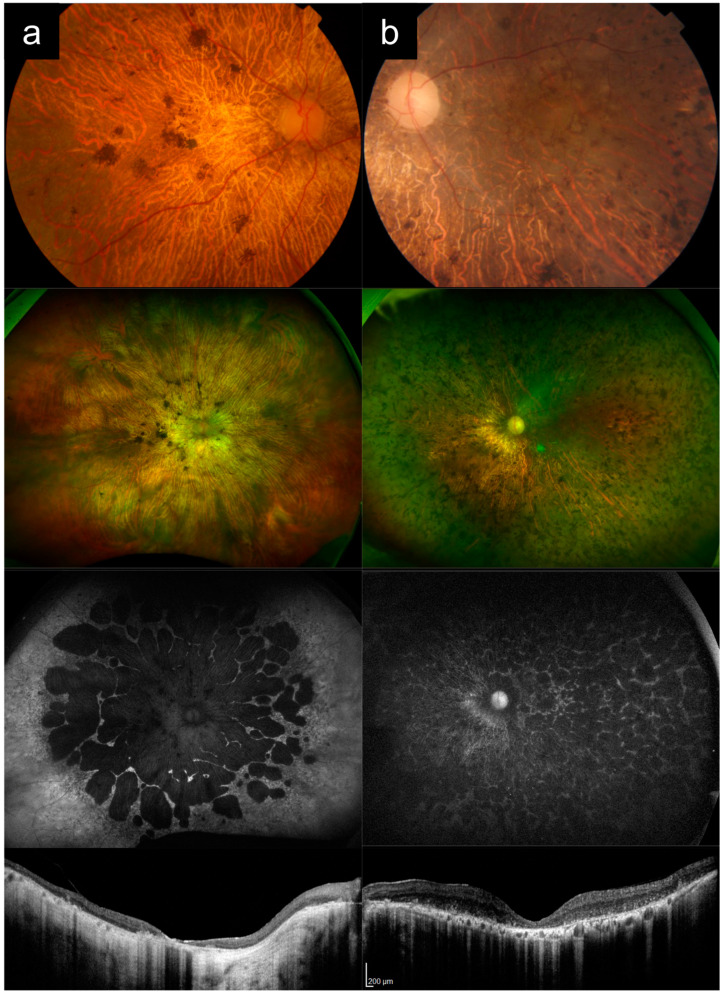
Cases 5a versus 5b: Severe, early-onset Stargardt disease (**a**) versus late-stage retinitis pigmentosa (**b**). Note the similar appearances of the posterior pole on color photography, with widespread diffuse atrophy, arteriolar attenuation, and pigment clumps. Ultrawidefield imaging shows that, in Case 5a, the distribution of disease is more central, with extensive macular involvement and centrifugal disease progression, which is more characteristic of *ABCA4*-associated retinal degeneration. In contrast, with retinitis pigmentosa the disease progression is centripetal. These cases can also be distinguished in terms of clinical history.

Case 5a had bilateral blurring of vision and hemeralopia from around 16 years old. Later, in his 20 s, he also developed nyctalopia. He had two siblings who were similarly affected, and no other known family history, consistent with AR inheritance. Figure 5a shows imaging of his right eye in his 60s. VA in the right eye was count fingers (CF) at the time. CFP imaging of the posterior pole shows widespread diffuse RPE and choroidal atrophy, with arteriolar attenuation and pigment clumps. UWF imaging better demonstrates that the pattern of RPE and choroidal atrophy is nummular, with confluence around the posterior pole. OCT imaging shows diffuse outer retinal, RPE, and choroidal atrophy. Genetic testing and segregation analysis revealed two causative *ABCA4* variants in trans (Table 1).

Case 5b presented first with nyctalopia in his late teens. Nyctalopia, peripheral vision and eventually central vision worsened progressively. He had consanguineous parents, and no other affected members of the family. Figure 5b shows imaging of the left eye from his 60s. VA in the left eye was hand motion (HM) at the time. CFP imaging shows widespread diffuse RPE atrophy with some possible narrow islands of relative sparing in the supertemporal macula, severe arteriolar attenuation, and bony spicule hyperpigmentation and clumps. UWF imaging shows that the atrophy is nummular and pan-fundal. OCT imaging shows diffuse outer retinal and RPE atrophy with choroidal thinning. Genetic testing analysis showed that this individual was homozygous for *REEP6* c.517+5G>T.

Cases of severe, early-onset Stargardt disease such as Case 5a can be frequently misdiagnosed as late-stage RP (or even choroideremia), based on widespread RPE and choroidal atrophy, arteriolar attenuation, and pigment clumping. Accurate clinical differentiation between these two entities can be helpful, as the genetic testing hypotheses for them are starkly different. An *ABCA4*-RD phenotype is typically a single-gene hypothesis, whereas RP can be caused by a very large number of candidate genes. Useful distinguishing features between the two are: (1) the clinical history for *ABCA4*-RD typically starts with central vision loss first and early in the disease process, whereas for advanced RP the presenting symptoms is nyctalopia and peripheral field loss, with central vision loss only late in the disease; (2) careful clinical examination (or UWF imaging) will usually show that *ABCA4*-RD has a more central distribution (even in late-stage disease), with extensive macular involvement, and centrifugal disease progression, whereas RP has a centripetal progression [24]; (3) peripapillary sparing, when present, points towards *ABCA4*-RD. However, as Case 5a illustrates, this is not always present, and, in fact, peripapillary sparing has been reported to be absent in about 2 to 7% of cases [25]; (4) pigment in *ABCA4*-RD is typically more prevalent in larger round clumps, whereas, in RP, it is usually more elongated and bony spicule-like; (5) if the pedigree suggests an inheritance pattern other than AR, this points away from *ABCA4*-RD, though one should always keep in mind the possibility of pseudo-dominant inheritance.

### 3.6. Cases 6a versus 6b: Severe, Early-Onset ABCA4-RD versus Leber Congenital Amaurosis

**Figure 6:**
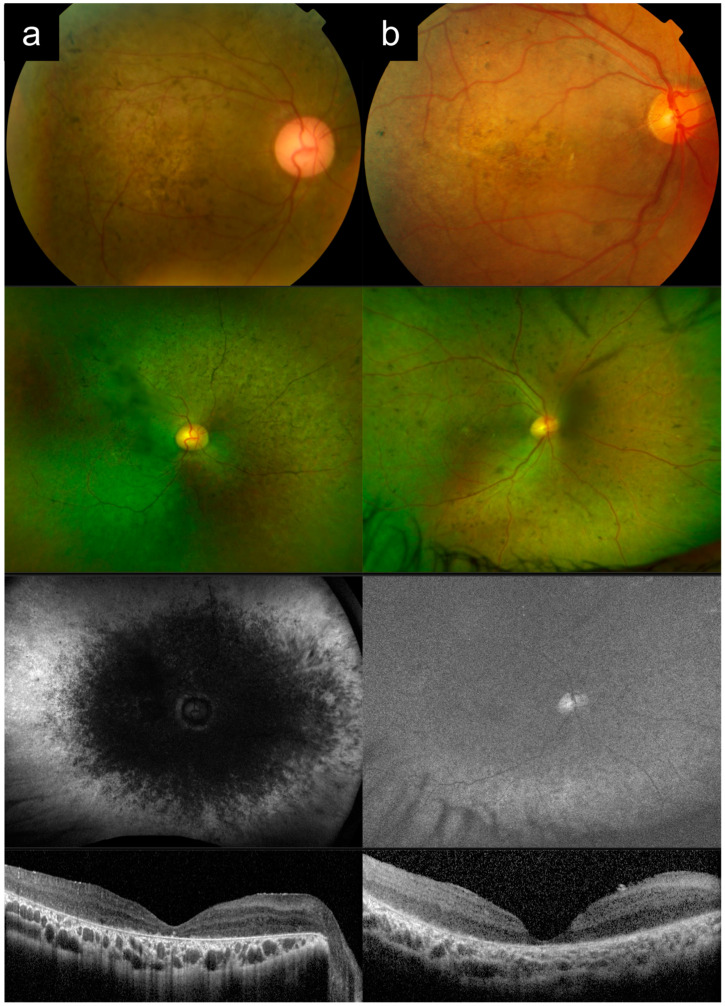
Cases 6a versus 6b: Severe, early-onset *ABCA4*-associated retinal degeneration (**a**) versus *CRB1*-associated Leber congenital amaurosis (LCA) (**b**). Note the similar appearances on color photography, with widespread diffuse atrophy and scattered pigment clumps. The cases also look similar on optical coherence tomography with diffuse outer retinal loss, inner retinal thickening, and loss of retinal laminations. The centrifugal distribution and peripapillary sparing on autofluorescence imaging in Case 6a points towards *ABCA4*-related retinal degeneration, while the relative lack of arteriolar attenuation in Case 6b points towards *CRB1*-associated LCA. These cases can also be distinguished clinically based on age of onset and presence or absence of nystagmus.

Case 6a presented with bilateral poor vision from 5 or 6 years old. At 7 years old, BCVA was 6/45 and 6/18 in the right and left eyes, respectively. She had no nystagmus. Vision progressively worsened over time. Figure 6a shows imaging of her right eye from her late teens. VA was HM in both eyes at this time. CFP and UWF imaging show diffuse widespread granular RPE atrophy with a yellow appearance to the fundus, scattered pigment clumps and significant arteriolar attenuation. UWF AF imaging shows that the atrophy is more central and centrifugal in distribution, with clear peripapillary sparing. OCT imaging showed diffuse outer retina and RPE atrophy, with mild diffuse thickening of the inner retinal layers, and mild loss of inner retinal lamination temporally. Genetic testing revealed two causative *ABCA4* variants in this patient (Table 1).

Case 6b presented with nystagmus, a divergent squint and poor vision noted in the first 3 months of life. At 1 year old, VA was about 6/120 and 6/200 in the right and left eyes, respectively. Figure 6b shows imaging of the right eye from 7 to 14 years of age. Visual acuity in the right eye was light perception (LP) at that time. CFP and UWF imaging show diffuse widespread granular RPE atrophy with a yellow appearance to the fundus and scattered pigment clumps. OCT imaging showed diffuse outer retinal and RPE atrophy, along with diffuse thickening and loss of distinct inner retinal lamination. Genetic testing disclosed a pathogenic and likely pathogenic *CRB1* allele, c.3153G>A p.W1051X and c.3493T>C p.C1165R.

Case 6a illustrates that a severe, early-onset *ABCA4*-RD can occasionally mimic Leber congenital amaurosis (LCA), and in this case particularly with LCA caused by *CRB1* variants. The *ABCA4*-RD case demonstrated widespread diffuse granular RPE atrophy, pigment clumps, and some mild inner retinal thickening and loss of lamination, which are more typically hallmarks of *CRB1*-associated disease. In this case, the key distinguishing features are the age of onset of visual loss, nystagmus, and the distribution of disease (Table 2). The onset later in life around 5 or 6 years of age, the absence of nystagmus, and the centrifugal distribution and peripapillary sparing (evident on UWF AF imaging) point towards *ABCA4*-RD rather than LCA. Some other potential features that are more consistent with *CRB1*-associated LCA are the relative lack of arteriolar attenuation (in relation to the severity of disease), and the more significant loss of retinal lamination on OCT. Other features which tend to occur in *CRB1*-associated disease (which are absent in this case) are periarteriolar preservation of the RPE, schitic change in the retina, and secondary Coat’s-like vascular exudation.

## 4. Discussion

The broad range of phenotypic diversity that can result from pathogenic mutations in *ABCA4* is remarkable. The *ABCA4* gene, located on chromosome 1, encodes for an ATP-binding cassette transporter in the outer segments of photoreceptors that participates in the visual cycle [16]. Reduction in *ABCA4* protein function from pathogenic mutations results in the accumulation of cytotoxic A2E and other bisretinoids in the retinal pigment epithelium (RPE), which causes RPE and photoreceptor loss [16]. It is thought that the phenotypic manifestations of pathogenic *ABCA4* mutations depend in large part on the amount of residual *ABCA4* protein function, as well as other genetic modifiers and as-yet undefined factors that affect susceptibility of cone photoreceptors and other retinal cell types to damage and loss [16].

In this series of selected *ABCA4*-RD cases, we present a range of phenotypes including “classical” Stargardt disease, fundus flavimaculatus, bull’s eye maculopathy without flecks, mild late-onset Stargardt disease mimicking pattern dystrophy, late-onset Stargardt disease mimicking dry AMD with geographic atrophy, and severe, early-onset disease mimicking late-stage RP, choroideremia and LCA. Each of these phenotypes has their own differential diagnoses and phenocopies, as well as important distinguishing features. Nevertheless, a few key distinguishing features across most of cases are the presence of peripapillary sparing, macular involvement and centrifugal distribution, and a recessive pedigree (Table 2). The presence of these features either in isolation or in combination should prompt an astute diagnostician to consider the possibility of *ABCA4*-RD.

This series of *ABCA4*-RD cases and their associated phenocopies is far from exhaustive. There are other reported phenotypes of *ABCA4*-RD and other important phenocopies not described within this series. Notable omissions include “Stargardt-like” macular dystrophies caused by *ELOVL4* and *PROM1* mutations (which have previously been termed STGD3 and STGD4, respectively), as well as other macular dystrophies or CORD caused by mutations in *BEST1*, *MT-TL1* (and other maternally inherited diabetes and deafness (MIDD) genes), *RDH12* and *CRX*, among others [16]. Another important though uncommon phenotype of *ABCA4*-RD that has been described is the optical gap phenotype, that presents on OCT imaging with a subfoveal lucent optically empty space and absent EZ line [26]. Potential phenocopies of the optical gap appearance on OCT imaging include achromatopsia (associated with *CNGA3* or *CNGB3* mutations), cone dystrophy (associated with *GUCY2D* or other genes), *RP1L1*-associated occult macular dystrophy, and acquired diseases such as solar retinopathy [26,27]. Finally, another important pair of conditions that present a major diagnostic dilemma is that of early-onset Stargardt disease and *CLN3*-associated Batten disease. Usually, this presents retinal specialists with an important diagnostic dilemma when faced with a child presenting between 5 and 10 years old with bilateral visual loss due to macular dystrophy [28]. *ABCA4*-RD and Stargardt disease is confined to the retina, but *CLN3*-associated Batten disease is a devastating diagnosis because of the systemic and neurological manifestations that typically result in a short life expectancy. Key distinguishing features of *CLN3*-associated Batten disease in this context are rapid visual loss, loss of color vision, optic disc pallor, loss of inner retinal laminations on OCT, abnormal dark-adapted full-field ERG responses, and the presence of vacuolated lymphocytes on a peripheral blood film [28,29].

In summary, we present a series of informative *ABCA4*-RD cases, along with their associated phenocopies that frequently mimic *ABCA4*-RD. Ultimately, most of these cases will require genetic testing confirmation, but the clinical index of suspicion for *ABCA4*-RD is important to minimize unnecessary diagnostic work-up, to narrow the genetic testing hypothesis, and to allow accurate, timely diagnosis for patients.

## Figures and Tables

**Table 1:** Presenting characteristics and genetic variant information for *ABCA4*-associated retinal degeneration in Cases 1a to 6a.

Case	Sex	Age of Onset (Years)	Presenting Symptom(s)	Causative Genetic Variants	ACMG Classification
1a	M	15	Poor central vision	*ABCA4* c.3328+1G>A *ABCA4* c.6193G>C p.D2065H	Likely pathogenic Likely pathogenic
2a	M	20	Difficulty reading	*ABCA4* c.3385C>T p.R1129C *ABCA4* c.1715G>A p.R572Q *ABCA4* c.4149delT p.F1383fs	Pathogenic Likely pathogenic Pathogenic
3a	F	57	Asymptomatic (incidental finding)	*ABCA4* c.1804C>T p.R602W *ABCA4* c.71G>A p.R24H	Pathogenic Pathogenic
4a	F	51	Poor central vision	*ABCA4* c.4217A>G p.H1406R *ABCA4* c.71G>A p.R24H	Pathogenic Pathogenic
5a	M	16	Poor vision Hemeralopia	*ABCA4* c.1712T>G p.I571S *ABCA4* c.885delC p.D295fs	Likely pathogenic Pathogenic
6a	F	5–6	Poor vision	*ABCA4* c.6642_6669dup p.S2224Pfs*36 *ABCA4* c.3523-2A>G	Likely pathogenic Pathogenic

**Table 2:** Key distinguishing features between *ABCA4*-associated retinal degeneration and phenocopies.

Cases	Features Favoring *ABCA4*-RD	Features Favoring Phenocopy
1a versus 1b: Typical adolescent-onset Stargardt disease versus *PRPH2*-associated macular dystrophy	Younger age of onset Peripapillary sparing Autosomal recessive inheritance	Older age of onset No peripapillary sparing Autosomal-dominant inheritance
2a versus 2b versus 2c: Stargardt disease without flecks versus *GUCY2D*-associated cone dystrophy versus hydroxychloroquine toxicity	Prominent central vision loss Macular dysfunction on electroretinography Autosomal recessive inheritance	Prominent glare and hemeralopia (2b) Diminished full-field cone responses (2b) Autosomal-dominant inheritance (2b) Drug history (2c)
3a versus 3b: Fundus flavimaculatus versus autosomal-dominant pattern dystrophy	Peripapillary sparing Autosomal recessive inheritance	Prominent pigment clumps within large yellow flecks Autosomal-dominant inheritance
4a versus 4b: Late-onset Stargardt disease versus age-related macular degeneration	Yellow flecks more elongated, meet at obtuse angles, creating pisciform, branching or reticular patterns Flecks hyper- or hypo-autofluorescence Nasal edge of atrophy concave to disc (due to peripapillary sparing) Island of central foveal sparing	Yellow drusen more round Drusen show less prominent autofluorescence abnormality Nasal edge of atrophy convex to disc Shallow irregular pigment epithelial detachment
5a versus 5b: Severe, early-onset Stargardt disease versus late stage retinitis pigmentosa	Central vision loss first and early extensive macular involvement with centrifugal progression Peripapillary sparing Pigment in larger round clumps Autosomal recessive inheritance	Nyctalopia and peripheral field loss early, with central vision loss late Centripetal progression Pigment more elongated and bony spicule-like Various inheritance patterns
6a versus 6b: Severe, early-onset Stargardt disease versus Leber congenital amaurosis	Later age of onset No nystagmus Centrifugal distribution and peripapillary sparing	Early age of onset (shortly after birth) Nystagmus

## Data Availability

The data presented in this study are available on request from the corresponding author. The data are not publicly available due to privacy restrictions.
